# Draft Genome Sequence of *Streptomyces* sp. Strain PAM3C, a Prospective Probiotic Agent Isolated from the Gut of the Bay Scallop, Argopecten purpuratus (Lamarck, 1819)

**DOI:** 10.1128/MRA.00640-21

**Published:** 2021-09-23

**Authors:** Wilbert Serrano, Raul M. Olaechea, Ulrike I. Tarazona

**Affiliations:** a Molecular Microbiology and Bacterial Genomics Laboratory, Universidad Científica del Sur, Lima, Perú; Indiana University, Bloomington

## Abstract

Several bacterial genera with antagonistic activity against pathogenic organisms have been isolated in pure culture from the gut of the Peruvian scallop, Argopecten purpuratus. One strain shows a broad inhibitory activity against aquaculture pathogenic bacterial strains. In this communication, we announce the draft genome sequence of *Streptomyces* sp. strain PAM3C.

## ANNOUNCEMENT

As part of a study of the microbiome of the Peruvian scallop (Argopecten purpuratus), one *Streptomyces* strain, PAM3C, was isolated in pure culture and was phenotypically characterized. This strain showed strong *in vitro* antagonistic activity against aquaculture pathogenic bacterial strains (1). Here, we present the draft genome sequence of the isolated organism.

Strain PAM3C was isolated from the gut of adult specimens of *A. purpuratus* collected in Independence Bay, Perú (14.2356 S, 76.1920 W). Single colonies were grown at 30°C for 5 to 8 days on Zobell marine agar 2816 (HiMedia, India) and were identified as *Actinobacteria* by the colony morphology and microscopic observation. Further characteristics, i.e., pigment production, aerial mycelia, and substrate mycelium formation, were determined by the use of International Streptomyces Project medium (2) under the conditions described above and examined according to reference 3. Genomic DNA from strain PAM3C was extracted following a chemical method which includes protein inactivation with SDS/proteinase K, polysaccharide precipitation with high-salt CTAB (cetyltrimethylammonium bromide), and selective DNA precipitation with cold isopropanol (4). Libraries were prepared using the TruSeq Nano library preparation kit and sequenced using the Illumina platform through a NovaSeq 6000 machine by Macrogen Inc. sequencing service (Seoul, South Korea). The system produced paired-end reads of 150 bp with >90% having Phred quality scores of at least Q30. Prior to assembly, the raw sequences were trimmed using the CLC Genomic Workbench v20.0.4 package (CLC bio/Qiagen) by removing reads with a quality score limit of 0.05 and sequences with lengths below 20 and allowing a maximum number of ambiguities of 2. The number of reads after trimming was 30,850,759. Afterward, *de novo* genome assembly was performed with the CLC package using the Bruijn-based assembly method with a word size of 20 and discarding contigs of <200. The draft assembly consists of 44 contigs with an average length of 174,226 bp. The *N*_50_ value of the assembly was 353,404 bp, with a G+C composition of the DNA of 72.3 mol% and a genome size of 7,665,955 bp. The genome sequence was annotated using the Prokaryotic Genome Annotation Pipeline (PGAP) from the National Center for Biotechnology Information (NCBI) (5). The system identified 6,849 coding sequences, of which 6,632 were predicted to encode hypothetical proteins, and 69 predicted noncoding RNAs. The presence of pathogenesis-related genes and antibiotic resistance genes was evaluated using the PathogenFinder v1.1 online tool of the Center for Genomic Epidemiology (https://cge.cbs.dtu.dk/services/PathogenFinder/) (6) and the Resistance Gene Identifier (RGI) v5.1.0 (https://card.mcmaster.ca/analyze/rgi) (7), respectively.

The 16S rRNA gene sequence of strain PAM3C showed an identity of >99% with the species Streptomyces variabilis and Streptomyces griseoincarnatus. An overall similarity comparison of orthologous fragments of genome sequences confirmed this close relationship (Table 1) and was calculated using the OrthoANI v0.93.1 algorithm of the EzBioCloud Web server (8). Further, an antiSMASH v6.0.1 search revealed the presence of 21 biosynthetic gene clusters, among them four lanthipeptide-encoding genes in strain PAM3C that were absent from the genomes of closely related *Streptomyces* species. Moreover, no pathogenesis- or resistance-related gene sequences were found within the assembled genome sequence of strain PAM3C.

**TABLE 1:** Orthologous average nucleotide identity comparison between strain PAM3C and closely and distantly related *Streptomyces* species

*Streptomyces* species	Ortho-ANI^*a*^ with PAM3C (%) (8)	Genome length (bp)	Avg length aligned with PAM3C (bp)	Coverage aligned with PAM3C (%)	GC content (%)
Strain PAM3C	100	7,601,040	7,601,040	100	72.31
Streptomyces variabilis	98.2	7,312,380	5,074,114	66.76	72.35
Streptomyces griseoincarnatus	98.18	7,385,820	4,989,794	65.65	72.12
Streptomyces griseoflavus	86.14	7,543,920	3,526,684	46.4	72.27
Streptomyces coelicolor	82.9	8,666,940	3,203,278	42.14	72.12
Streptomyces griseoaurantiacus	81.21	7,379,700	2,608,119	34.31	72.79
Streptomyces rochei	81.61	8,364,000	2,550,108	33.55	71.7

a OrthoANI, orthologous average nucleotide identity.

### Data availability.

The 16S rRNA gene and whole-genome shotgun sequences have been deposited at DDBJ/ENA/GenBank under accession numbers MG195145 and GCA_018966745.1, respectively. The raw sequencing data are available in the Sequence Read Archive (SRA) database under accession number SRR14800108. The associated BioProject accession number is PRJNA737002.
